# Evaluation of frontal QRS-T angle values in electrocardiography in patients with chronic rhinosinusitis

**DOI:** 10.1186/s12872-023-03175-1

**Published:** 2023-03-27

**Authors:** Sabri Abus, Mehtap koparal, Hakan Kaya, Olga Bayar Kapıcı, Mehmet Hakan Tasolar, Hakan Tibilli

**Affiliations:** 1 Department of Cardiology, Adiyaman University Education and Research Hospital, Adiyaman, Turkey; 2 Department of Otorhinolaryngology, Adiyaman University Education and Research Hospital, Adiyaman, Turkey; 3 Department of Radiology, Adiyaman University Education and Research Hospital, Adiyaman, Turkey; 4 Department of Cardiology, Inonü University, Malatya, Turkey; 5 Department of Cardiology, Adıyaman Univesity, 416100 Adıyaman, Turkey

**Keywords:** Chronic rhinosinusitis, Neutrophil to lymphocyte ratio, Frontal QRS-T angle

## Abstract

**Background:**

Chronic Rhinosinusitis (CRS) refers to inflammation of the paranasal sinuses and nasal mucosa. Electrocardiographic indicators of ventricular repolarization have been shown to correlate with systemic inflammation parameters. Recently, the frontal QRS-T (fQRS-T) angle has been accepted as a new indicator of ventricular depolarization and repolarization heterogeneity. The (fQRS-T) angle is recommended in predicting the risk of malignant ventricular arrhythmia. In this study, we aimed to evaluate the ventricular arrhythmia potential in patients with chronic rhinosinusitis by examining the relationship between fQRS-T angle on ECG and inflammation markers.

**Methods:**

Inflammatory markers as well as electrocardiographc (ECG) f(QRS-T) angle, QRS duration, QT interval and corrected QT interval were examined in 54 patients with CRS versus 56 healthy control subjects.

**Results:**

The f(QRS-T) angle was significantly higher in CRS patients than in healthy controls (p < .001). The neutrophil-to-lymphocyte ratio (NLR), platelet-to-lymphocyte ratio (PLR) and monocyte-to-lymphocyte ratio (MLR) were significantly higher in CRS patients compared to healthy controls (p < .001, for all). Based on correlation analysis, NLR and f(QRS-T) angles were highly correlated (r = .845, p < .001), and according to the results of linear regression analysis, NLR was independently associated with the f(QRS-T) angle (t = 5.149, Beta = 0.595, p = < 0.001).

**Conclusion:**

Both f(QRS-T) angle and NLR are significantly increased in CRS patients compared to healthy controls, with increases in NLR also independently associating with increases in f(QRS-T) angle. While the increases in f(QRS-T) angle did not result in clinically alarming absolute values for f(QRS-T), CRS patients might nonetheless be at relatively higher risk for malignant cardiac arrhythmias.

## Introduction

Rhinosinusitis refers to inflammation of the paranasal sinuses and nasal mucosa. Rhinosinusitis presents at least two symptoms inclusive of post-nasal drip, mucopurulent runny nose, nasal congestion, and facial pain associated with the affected sinuses [1]. If these symptoms persist for more than 12 weeks, chronic rhinosinusitis (CRS) is diagnosed [2].

Chronic inflammation has been associated with atherosclerosis, endothelial dysfunction, and cardiovascular diseases. Endothelial dysfunction is responsible for atherosclerosis initiation and continuation [3]. Previous studies have shown that the risk of acute myocardial infarction is higher in CRS patients [4].

Stromal neutrophil invasion is implicated in the pathogenesis of CRS. It has been reported that the increase in the neutrophil count alone is insufficient to indicate inflammation, but the neutrophil-to-lymphocyte ratio (NLR) is more significant in chronic inflammation. It has been shown that the platelet-to-lymphocyte ratio (PLR) is increased in patients with CRS [5]. C-reactive protein (CRP) is an essential indicator of inflammation and is widely used to assess acute infections. It has been shown that the CAR value, which is the ratio of CRP to albumin, is a significant predictor of mortality and morbidity [6]. It has been reported that the monocyte-to-lymphocyte ratio (MLR) is a significant predictor of mortality in head and neck cancers [7]. Monocyte to high-density lipoprotein (HDL) ratio (MHR) is used as an indicator of atherosclerosis [8].

Frontal QRS-T (f(QRS-T)) angle, the absolute angular difference between the directions of ventricular depolarization (QRS axis) and repolarization (T axis), is a relatively new indicator of ventricular depolarization and repolarization heterogeneity. Since the QRS and T axes are generally accessible from the computerized records of most 12-lead ECG devices, the f(QRS-T) angle can be readily computed based on the absolute directional difference between the two axes. Earlier studies have demonstrated the prognostic value of the f(QRS-T) angle in numerous cardiovascular disorders. Moreover, notably increased f(QRS-T) angles have been related to increased risk for arrhythmia and sudden cardiac death [9].

The aim of this study was to examine how the f(QRS-T) angle relates to inflammatory markers in patients with CRS versus in healthy controls.

## Material and method

### Study sample

This was a prospective, cross-sectional study. Approval was acquired from the local Ethics Committee of Adiyaman University (approval no:2022/1–25). Informed consent was obtained from all subjects, in accordance with the Helsinki Declaration. Patients aged 18–45 years with a diagnosis of CRS followed by the otolaryngology outpatient clinic were evaluated in the cardiology outpatient clinic. In the patients’ paranasal sinus computed tomographic (CT) evaluations, chronic sinusitis was diagnosed with findings such as thickening of the sinus mucosa, sclerotic thickening of the bone, intrasinus calcification air-fluid level, and osteomeatal obstruction. Patients with diabetes mellitus (n = 2), hypertension (n = 3), systemic lupus erythematosus (n = 1) or asthma (n = 1) with chronic sinusitis were not included in the study. The study also did not include those with a family history of coronary artery disease, dysrhythmia, or heart valve disease (n = 4), nor or those with iron deficiency anemia or acute infection as assessed by laboratory results (n = 3). Those with signs of acute sinusitis, regular use of intranasal corticosteroids, or with nasal polyps or septal deviations in paranasal CT and clinical examination (n = 6) were also excluded from the study. As a result, 24 male and 30 female patients between 18 and 45 were included in the study.

The healthy control group included people who applied to the cardiology outpatient clinic for employment checkups, military service examinations, driver’s licenses who did not have any known disease. A total of 37 female and 19 male healthy controls were included.

### Electrocardiogram examination

Each patient’s 12-lead electrocardiogram (ECG) was analyzed blindly by two independent cardiologists. A supine 12-lead ECG recording (50 mm/s, 10 mm/mV) was obtained using a CardioFax S device (Nihon Kohden, Tokyo, Japan). Resting heart rate was quantified using ECG data. Caliper and magnifying glass were utilized to lessen quantification mistakes. QRS duration was defined as the time from the beginning to the end of the QRS complex. The QT interval was calculated as the time from the beginning of the QRS complex to the end of the T wave. The QT interval was corrected for heart rate using Bazett’s method [10].

The QRS and T wave axes were available in the mechanized records of the ECG machine. The f(QRS-T) was computed from these axes as the absolute difference between the frontal plane QRS axis and the frontal plane T axis. The axes themselves were also determined by subtracting 360° whenever the given direction exceeded 180° on a 0-360° scale.

### Laboratory analyses

Venous blood specimens were analyzed upon admission to the hospital. Total white blood cell count and neutrophil, lymphocyte, monocytes, eosinophil, and basophil counts were measured using a device (CELL-DYN Ruby; Abbott Diagnostics, Abbott Park, IL) and given as x 10^3^ cells/mm^3^. Hemoglobin, hematocrit, and thrombocyte numbers were also measured. Creatinine, urea, andCRP levels were analyzed using biochemistry kits (Abbott Diagnostics) and an Architect c8000 Chemistry System (Abbott Diagnostics) machine. Total cholesterol, fasting triglyceride, HDL, low-density lipoprotein (LDL), and fasting blood glucose plasma concentrations were measured by the enzymatic chemical clearing process operating Cobas 6000 (Roche Diagnostics GmbH, Mannheim, Germany). NLR, PLR, MLR and MHR were calculated.

### Statistical analysis

Statistical analyses were conducted in SPSS 26.0 for Mac (SPSS Inc., Chicago, IL). Categorical data were expressed as numbers and percentages. The conformity of the data to the normal distribution was evaluated with the Kolmogorov-Smirnov test. The mean and standard deviation values of the continuous data were given. Independent samples t-test and chi-square test were used to compare ECG and laboratory parameters between patients and controls. Pearson correlation analysis, linear and logistic regression analysis examined the relationship between inflammation and ECG parameters.A p value < 0.05 was considered statistically significant.

## Results

The comparison of age and gender information and ECG parameters in the CRS patients and healthy controls are shown in Table 1. No significant differences were found between the two groups with respect to age and gender. The QRS duration, QT interval and corrected QT intervalwere similar between the two groups (p > .05, for all). Heart rate was significantly higher in CRS patients, and the f(QRS-T) angle was significantly higher in CRS patients than in healthy controls (p = .034).

**Table 1:** Comparison of Sociodemographicand ECG Parameters of PatientswithChronicRhinosinusitisandHealthyControls

		CRS Patients (n = 54) M ± SD orN(%)	HC (n = 56) M ± SD orN(%)	X^2^or t value	p
**Age**		34.61 ± 6.47	33.02 ± 6.51	-1.286	.201^1^
**Gender**	**Female**	30 (55.6)	37 (66.1)	1.277	.258^2^
	**Male**	24 (44.4)	19 (33.9)		
**Heart Rate (bpm)**		83.59 ± 15.18	77.91 ± 12.43	-2.151	.034^1^
**QRS (msec)**		89.85 ± 9.06	87.5 ± 8.54	-1.401	.164^1^
**QT (msec)**		364.3 ± 30.24	366.61 ± 28.48	0.413	.681^1^
**QTc (msec)**		412.06 ± 24.26	405.86 ± 30.45	-1.178	.241^1^
**f(QRS-T)angle(** ^**0**^ **)**		49.5 ± 26.61	22.2 ± 17.12	-6.373	< .001^1^

^1^Independent t test wasused. ^2^Pearson chi-square test wasused. p < .05 wasaccepted as staticallysignificant

CRS: ChronicRhinosinusitis; HC: Healthy Control; QTc: Corrected QT interval; bpm: beatperminute; f(QRS-T): Frontal QRS-T

The comparison of laboratory parameters in CRS patients versus healthy controls are shown in Table 2. While the neutrophil count was significantly higher in the CRS patients, the lymphocyte count and albumin level were significantly lower in the CRS patients. NLR, PLR, and MLRwere significantly higher in the CRS patients (p < .001, for all).

**Table 2:** Comparison of LaboratoryParameters of PatientswithChronicRhinosinusitisandHealthyControls

	CRS Patients (n = 54) M ± SD orN(%)	HC (n = 56) M ± SD orN(%)	tvalue	p
**Hemoglobin (g/dL)**	14.76 ± 1.99	14.37 ± 2.11	-1.002	0.319
**Platelet(1/uL)**	245,527 ± 45,773	241,870 ± 58,265	− 0.365	0.716
**Neutrophil(10** ^**3**^ **/uL)**	5852.19 ± 1830.38	5017.73 ± 1733.90	-2.455	0.016*
**Lymphocyte(1/uL)**	1571.48 ± 636.16	2467.64 ± 773.81	6.622	< 0.001**
**Monocyte(1/uL)**	548.87 ± 227.65	538.41 ± 203.60	− 0.254	0.800
**Eosinophil(1/uL)**	127.06 ± 115.55	143.96 ± 112.64	0.777	0.439
**Basophil(1/uL)**	87.52 ± 44.79	91.25 ± 46.53	0.551	0.669
**Albumin(g/dL)**	3.95 ± 0.24	4.33 ± 0.48	0.102	< 0.001**
**CRP (mg/dL)**	0.27 ± 0.10	0.25 ± 0.25	− 0.759	0.449
**Total Cholesterol (mg/dL)**	178.44 ± 44.30	168.70 ± 39.70	-1.216	0.227
**HDL (mg/dL)**	63.77 ± 13.84	67.32 ± 16.73	1.210	0.229
**LDL (mg/dL)**	81.35 ± 30.81	75.44 ± 28.85	-1.024	0.308
**FastingTriglyceride(mg/dL)**	161.46 ± 143.74	126.64 ± 112.59	-1.417	0.159
**CAR**	0.07 ± 0.02	0.06 ± 0.07	− 0.998	0.320
**NLR**	4.08 ± 1.26	2.23 ± 1.08	-8.237	< 0.001**
**MLR**	0.40 ± 0.23	0.23 ± 0.13	-4.503	< 0.001**
**PLR**	178.80 ± 68.21	108.16 ± 49.74	-6.187	< 0.001**
**MHR**	8.78 ± 3.61	8.55 ± 3.93	-0.326	0.745

Independent t test wasused. p < .05 wasaccepted as staticallysignificant

CRS: ChronicRhinosinusitis; HC: Healthy Control; CRP: C-reactive protein; HDL: High densitycholesterol; LDL: LowdensityCholesterol; CAR: C-reactive protein/albuminratio; NLR: Neutrophil/Lymphocyteratio; MLR: Monocyte/Lymphocyteratio; PLR:Platelet/Lymphocyteratio; MHR: Monocyte/highdensitycholesterolratio

According to the correlation analysis of f(QRS-T) and inflammation parameters in patients, a positive and significant correlation was found between NLR, PLR, MLR and f(QRS-T) angle (Table 3).

**Table 3:** Correlation Analysis of InflammationMarkersandFrontal QRS-T Angle in ThePatientswithChronicRhinosinusitis

		f(QRS-T)Angle
**Age**	r	0.248
	p	0.71
**NLR**	r	0.845
	p	< 0.001**
**MLR**	r	0.639
	p	< 0.001**
**PLR**	r	0.673
	p	< 0.001**
**CAR**	r	− 0.113
	p	0.418
**MHR**	r	0.187
	p	0.175

Pearsoncorrelationanalyseswasused. p < .05 wasaccepted as statisticallysignificant

CAR: C-reactive protein/albuminratio; NLR: Neutrophil/Lymphocyteratio; MLR: Monocyte/Lymphocyteratio; PLR:Platelet/Lymphocyteratio; MHR: Monocyte/highdensitycholesterolratio

According to the applied linear regression analysis, the NLR and f(QRS-T) angle values were also highly related in CRS patients (ANOVA F:17.313, p < .001, Adjusted R^2^: 0.512) (Table 4).

**Table 4:** LinearRegression Analysis of Frontal QRS-T AnglebyInflammationMarkers in ThePatientswithChronicRhinosinusitis

	B	Beta	t	p	%95 CI	
					Lower	Upper
**Age**	0.305	0.089	1.321	0.189	− 0.164	0.553
**Gender**	3.897	0.086	1.213	0.228	-6.270	4.475
**NLR**	8.882	0.595	5.149	< 0.001	5.460	12.303
**MLR**	20.705	0.192	1.413	0.161	-8.367	49.776
**PLR**	0.007	0.026	0.197	0.844	− 0.066	0.080
**CAR**	11.167	0.028	0.395	0.693	-44.874	67.207
**MHR**	− 0.459	− 0.078	− 0.743	0.459	-1.686	0.767
**Constant**	15.310		1.546	0.125	-4.333	34.952

Lineer regressionanalyseswasused. p < .05 wasaccepted as staticallysignificant

NLR: Neutrophil/Lymphocyteratio;MLR: Monocyte/Lymphocyteratio; PLR:Platelet/Lymphocyteratio;CAR: C-reactive protein/albuminratio;MHR: Monocyte/highdensitycholesterolratio

## Discussion

The present study demonstrated that f(QRS-T) angles are statistically significantly greater in CRS patients than in age- and gender-matched controls. To our knowledge, this is the first study in the literature to evaluate f(QRS-T) angles in CRS patients.

Neutrophils are short-lived white blood cells involved in inflammation. It has been reported that maxillary sinus lavage neutrophil count is increased in CRS patients. Interleukin-8 has also been shown to activate neutrophils and potentially cause this neutrophil elevation [11]. Elevated NLR has been reported in many malignancies and diseases. In one study, NLR was shown to be higher in patients with Bell’s palsy, and improvement was less in patients with higher NLR values ​​[12]. In a study of CRS patients, NLR and PLR were higher in CRS patients with or without nasal polyposis [5]. In their study, Boztepe et al. found that NLR values ​​were not different in CRS patients without nasal polyposis compared to healthy controls, while PLR ​​was higher in CRS patients [13]. NLR has been associated with lower survival rates in patients with acute heart failure [14]. Mortality rates after ST elevation acute myocardial infarction are high in patients with elevated NLR [15]. In another study, NLR was high in patients with nasal polyposis [16].

It has been reported that there is an increased risk of atherosclerosis in chronic inflammation. However, in our study, MHR values ​​in CRS patients were not different from healthy controls. Neutrophils damage the vessel walls through the mediators they secrete, while lymphocytes exert an antiatherosclerotic effect. Therefore, elevated NLR is associated with impaired vascular health and an increased risk of cardiovascular disease [17]. This study found a positive correlation between f(QRS-T), an indicator of cardiovascular disease risk, and NLR.

Platelets trigger the inflammation process through the chemokines and cytokines they secrete. In addition, platelets cause an atherothrombotic process by forming a thrombus in response to plaque fragmentation. Platelet activation in these ways causes an increased risk of cardiovascular disease. It has been reported that the death rate from acute myocardial infarction is increased in patients with high platelet counts. PLR has been reported to be elevated in hypertension, venous thromboembolism, renal failure, and cancer [18].

Monocytes and monocyte-derived phagocytes play a role in the formation of atherosclerosis. Monocytes transform into macrophages after arterial wall invasion. Macrophages cause the rupture of atherosclerotic plaques through the proinflammatory cytokines they secrete and oxygen radicals’ contribution [19]. It is known that the lymphocyte count decreases in chronic inflammation. It has been reported that low lymphocyte count is an unfavorable predictor in the recovery period after acute myocardial infarction [20]. Although MLR is more frequently reported to be increased in malignancy and tuberculosis, it is thought that increased MLR may be associated with cardiovascular disease risk [21].

QRS-T angles can be calculated in two main ways: first in three-dimensional space, i.e., as the spatial QRS-T angle, either as a mean or a “peaks” spatial QRS-T angle [22]; and second more directly from standard 12-lead ECGs as the frontal plane projection f(QRS-T) angle [23]. If not immediately provided via a given ECG manufacturer’s software, computation of spatial QRS-T angle can be rather complex. However, the spatial peaks QRS-T angle can still be laboriously estimated from the conventional ECG [24]. In contrast, the f(QRS-T) angle can be more easily estimated by clinicians from the surface ECG by just calculating the absolute value of the directional difference between the frontal-plane QRS and T-wave axes that most ECG machines already automatically report. In addition, in some earlier research, it’s been suggesed that f(QRS-T) angle can be a reasonable clinical alternative for spatial QRS-T angle in risk estimation [25]. In healthy individuals, the myocardial depolarization and repolarization axes tend to have similar orientations. Thus, the f(QRS-T) angle tends to be narrower than < 45˚ [26]. A broader f(QRS-T) angle indicates a mismatch between ventricular depolarization and repolarization phases, and in patients with left ventricular systolic dysfunction after myocardial infarction, an f(QRS-T) value > 90˚ results in a less favorable prognosis [27]. The f(QRS-T) angle is a basic, reliable and readily accessible parameter. And based on a prior study of the luminal caliber of coronary arteries, for this study, we also considered any increase in f(QRS-T) > 45˚ as abnormal [26].

The increase in f(QRS-T) angle in CRS patients may result from chronic inflammation. A recent study showed that electrocardiographic indicators of abnormal ventricular repolarization correlate with systemic inflammation parameters such as high-sensitivity CRP and interleukin-6, And additionally escalated inflammatory activity, directly or through oxidative stress and apoptosis, can also potentially trigger malignant ventricular arrhythmias [28, 29]. In our study, a positive and significant relationship was also found between NLR, PLR, and MLR. In addition, as a result of linear regression analysis between these parameters, a statistically significant relationship was observed between NLR and f(QRS-T) angle.

Another reason for increased f(QRS-T) angle in CRS patients may be cardiac autonomic dysfunction due to anxiety and depression. Recent studies have shown that the frequency of depression and anxiety in CRS patients is significantly greater than in the average population [30]. Depression and anxiety have been further investigated for their long-period association with arrhythmia risk. This notion may echo the well-known impact of depression on long-period prognosis in patients with coronary artery disease [31]. In addition, depression has been researched more intensively due to its relationship with events like sudden cardiac arrest due to malignant cardiac arrhythmia [32]. Changes in cardiac autonomic neural tone and high sympathetic activity in the ventricular myocardium are linked with total repolarization distribution and increased arrhythmia risk [33]. Cardiovascular autonomic dysfunction, sympathetic dominance, and parasympathetic withdrawal are frequently proposed mechanisms linking arrhythmia risk to psychological distress. Grippo et al. studied a depressive phenotype in a rat model through stress induction, the components of which included exposures to continuous nighttime lighting, paired housing, and white noise. Rats exposed to such stress developed increased heart rate, decreased heart rate variability, and, more importantly, ventricular arrhythmia in response to aconitine infusion [34]. Carney and colleagues have shown that depressed patients in a post-myocardial infarction setting have more reduced heart rate variability, particularly in the very low frequency domain, compared to non-depressed patients during 24-hour ambulatory ECG monitoring [35]. Anxiety and depression may also cause an increase in f(QRS-T) angle and potentially contribute to the development of malignant cardiac ventricular arrhythmias in CRS patients due to autonomic nervous system dysfunction and increased sympathetic activity.

### Limitations

The present study has some limitations, including the small number of patients, the use of data from a single hospital, and the lack of any follow-up with respect to the occurrence of any malignant cardiac arrhythmias or sudden cardiac deaths in our patients.

## Conclusion

In conclusion, Both f(QRS-T) angle and NLR are significantly increased in CRS patients compared to healthy controls, with increases in NLR also independently associating with increases in f(QRS-T) angle. While the increases in f(QRS-T) angle did not result in clinically alarming absolute values for f(QRS-T), CRS patients might nonetheless be at relatively higher risk for malignant cardiac arrhythmias. However, more extensive and advanced multicenter studies in which CRS patients are followed for malignant cardiac arrhythmias are required to confirm the potential import of our findings.

## Data Availability

The datasets used and analyzed during the current study are available from the corresponding author on reasonable request.

## Abbreviations

CRS: Chronic Rhinosinusitis
f(QRS-T): Frontal QRS-T angle
ECG: Electrocardiogram
CT: Computed tomography
CRP: C-reactive protein
HDL: High-density lipoprotein
LDL: Low density lipoprotein
NLR: Neutrophil lymphocyte ratio
PLR: Platelet lymphocyte ratio
MLR: Monocyte lymphocyte ratio
